# Genotoxicity and Safety Pharmacology Studies of Indole Alkaloids Extract from Leaves of *Alstonia scholaris* (L.) R. Br.

**DOI:** 10.1007/s13659-020-00242-4

**Published:** 2020-04-30

**Authors:** Yun-Li Zhao, Min Su, Jian-Hua Shang, Xia Wang, Guang-Lei Bao, Jia Ma, Qing-Di Sun, Fang Yuan, Jing-Kun Wang, Xiao-Dong Luo

**Affiliations:** 1 State Key Laboratory of Phytochemistry and Plant Resources in West China, Kunming Institute of Botany, Chinese Academy of Sciences, Kunming, 650201 People’s Republic of China; 2 Yunnan Institute of Medical Material, Kunming, 650111 People’s Republic of China; 3 Key Laboratory of Medicinal Chemistry for Natural Resource, Ministry of Education and Yunnan Province, School of Chemical Science and Technology, Yunnan University, Kunming, 650091 People’s Republic of China; 4 Jiangsu Nhwa Pharmaceutical Co., Ltd, Xuzhou, 221009 People’s Republic of China

**Keywords:** *Alstoniascholaris*, Indole alkaloids extract, Genotoxicity, Safety pharmacology, Mice, Dogs

## Abstract

**Abstract:**

Indole alkaloids extract (IAAS) was prepared from leaves of *Alstonia scholaris* (L.) R. Br., an evergreen tropical plant widely distributed throughout the world. This plant has been used historically by the Dai ethnic people of China to treat respiratory diseases. This study evaluated the genotoxicity and safety pharmacology of IAAS to support clinical use. The bacterial reverse mutation (Ames) test, in vitro mammalian chromosomal aberration test, and in vivo mammalian erythrocyte micronucleus (MN) test were performed to evaluate genotoxicity. Mice were administered IAAS (240, 480, or 960 mg/kg bw) once orally to observe adverse central nervous system effects. Furthermore, beagle dogs were administered IAAS (10, 30, 60 mg/kg bw) once via the duodenum to evaluate its effects on the cardiovascular and respiratory systems. IAAS with or without S9-induced metabolic activation showed no genotoxicity in the Ames test up to 500 μg/plate, in the mammalian chromosomal aberration test up to 710 μg/mL, or in the MN test up to 800 mg/kg bw. No abnormal neurobehavioral effects were observed in mice following treatment with up to 960 mg/kg bw of IAAS. Moreover, blood pressure, heart rate, electrocardiogram parameters, and depth and rate of breathing in anesthetized beagle dogs did not differ among the IAAS doses or from the vehicle group. These data indicated that IAAS did not induce mutagenicity, clastogenicity, or genotoxicity, and no pharmaco-toxicological effects were observed in the respiratory, cardiovascular, or central nervous systems. Our results increased understanding of safety considerations associated with IAAS, and may indicate that IAAS is a possible drug candidate.

**Graphic Abstract:**

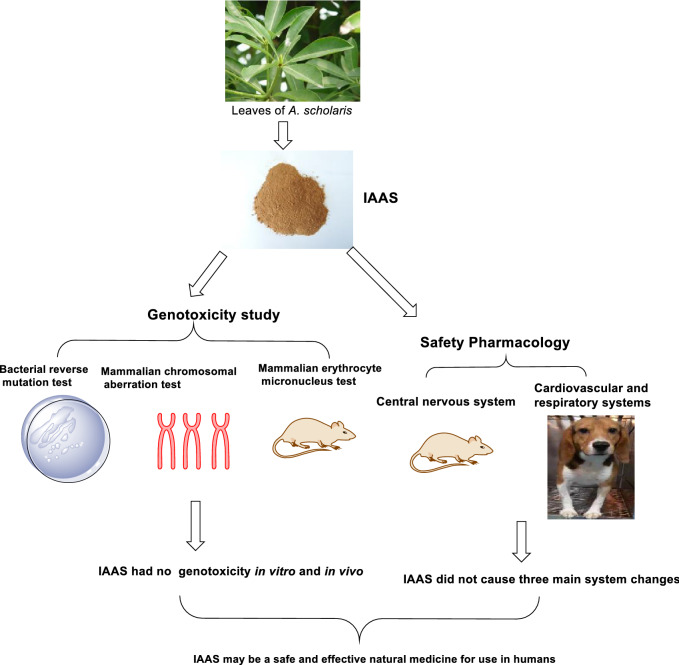

**Electronic supplementary material:**

The online version of this article (10.1007/s13659-020-00242-4) contains supplementary material, which is available to authorized users.

## Introduction

*Alstoniascholaris* (L.) R. Br., an Apocynaceae evergreen plant with white funnel-shaped flowers and milky sap, is widely found in tropical and subtropical mountains, and valley rainforests regions in Asia and Africa. This plant has significant medicinal value [1]. Preparations derived from different parts of the plant can be used as natural medicines or natural plant extracts for treatment of various human ailments. The ethyl acetate extract of the flowers has been shown to inhibit *Escherichiacoli* [2]. The bark is used as a tonic, an aphrodisiac, a febrifuge, an expectorant, an alternative, a carminative, a stimulant, an antiperiodic, and an astringent [3]. Furthermore, the bark has also been used to treat bowel complaints in India, abdominal pain, dysentery, and fever in Australia, and malaria [4]. The stem is used to treat fever with chills [5] and stem decoction can be used as an analgesic during labor, can relieve pain from contractions, and decrease labor [6]. The root of *A.Scholaris* is used as an expectorant, a stomachic, and to treat gum or dental problems [7]. The latex of this plant is used as an ingredient in chewing gum [8] and the aqueous extract of the latex is used to treat tuberculosis [9] and asthma, [10], and can be used as an abortifacient [11]. The leaves are traditionally used for management of respiratory ailments in Dai medicine practiced in China [12]. For years, our group has extensively studied the characteristics, and the differences in constituents, of different parts of *A.scholaris* [13–32]. Among the compounds isolated, eight monoterpenoid indole alkaloids from *A. scholaris* have been selected as “hot off the press” compounds, as specified in *NaturalProductsReport* (Supplementary materials, Table S1). Moreover, 16 *Alstonia* alkaloids were synthesized by chemists following structural identification, and studies of their bioactivities were published (Supplementary materials, Table S2).

Identification of chemical constituents has allowed for further characterization of the activities of indole alkaloids by our team. Shang et al. reported that the ethanolic extract *A. scholaris* leaves eased cough, diminished phlegm inflammation, reduced acute exacerbation of asthma [33], and alleviated pain and inflammation [34]. Moreover, Zhao et al. showed that the indole alkaloids extract (IAAS) of *A. scholaris* leaves could inhibit airway inflammation and alleviate airway damage in Sprague–Dawley rats induced by lipopolysaccharide [35]. These findings led to further studies of *A. scholaris* leaves. Relief of ovalbumin-induced allergic asthma symptoms by IAAS was attributed to scholaricine, 19-epischolaricine, vallesamine, and picrinine (Supplementary materials, Fig. S1A) [36]. IAAS also protected against LPS-induced post-infectious cough in vivo [37]. We further explored the mechanism of action of these molecules and found that β2 adrenergic receptors were activated [38], and nuclear factor-κB bioactivity was inhibited, by these alkaloids in vitro [39]. Cao et al. showed that these four alkaloids were metabolized by hydroxylation and glucuronidation reactions in rats [40]. These findings led to the hypothesis that IAAS from *A. scholaris* could be used as a novel natural treatment for respiratory diseases, and may provide lasting benefits to individuals with asthma, post-infection cough, and acute tracheal bronchitis.

Although *A. Scholaris* leaves are widely used medicinally, toxicological data is scarce. Baliga et al*.* evaluated acute and 30-day repetitive intraperitoneal injection toxicity of the hydroalcoholic extract of *A. scholaris* stem bark in mice and rats. The results showed that 120 mg/kg bw bark extract treatment for up to 30 days did not induce any marked changes. However, the animals exhibited lethargy and ducking in response to 240 mg/kg bw [41]. In addition, administration of 360 and 480 mg/kg bw bark ethanol extract to pregnant Swiss albino mice resulted in congenital abnormalities such as syndactyly, bent tails, and developmental delays in newborn mice [42]. Furthermore, the repeat-dose oral toxicity of the methanol extract of the bark was evaluated in Sprague–Dawley rats for 28 consecutive days, and changes in hematological compositions and end-organ damage to the liver were observed in response to doses of 500 and 1,000 mg/kg bw [41]. These studies focused exclusively on toxicity of stem bark. However, toxicological data for *A. scholaris* leaves is scarce. Therefore, it is important to evaluate genotoxicity and safety pharmacology of *A. scholaris*. We performed a systematic toxicological evaluation to provide the foundation for a possible comprehensive traditional preclinical study and to determine the safety of IAAS.

This study was performed at the Drug Safety Evaluation Center of Yunnan Institute of Medical Material (Yunnan, China) in compliance with the Testing Guidelines for Safety Evaluation of Drugs (Notification [S] GPT1-1, issued by the China Food and Drug Administration (CFDA) under Good Laboratory Practice Regulations [43].

## Results

### Genotoxicity Studies

#### Bacterial Reverse Mutation Test

A Good Laboratory Practice (GLP)-compliant bacterial reverse mutation study was conducted to investigate the mutagenic potential of IAAS using five different histidine-requiring strains of *S. typhimurium*, each responding to distinct classes. No precipitation or specific drug-dependent cytotoxic effects of IAAS were observed in this experiment. The number of revertant colonies in the vehicle group was within the range of laboratory historical data, and the positive mutagenic agents (NaN3, 2-AF) caused significant increases in the number of revertant colonies in each of the tested strains, which indicated that the detection system was valid (Table S3). No significant increases in revertant colonies were observed following exposure to IAAS at any concentration level in any of the five test strains. These results were independent of the presence of S9 metabolic activation. These results indicated that IAAS was not mutagenic.

#### In Vitro Mammalian Chromosomal Aberration Test

As shown in supplementary materials Table S4, the IC_50_ of IAAS was 704 μg/mL on Chinese hamster lung (CHL) cells. Therefore, 710, 355, 178, 89, and 44 μg/mL concentrations were used in experiments A and B. No aberrations were observed in the vehicle group. In contrast, the chromosomal aberration rates in response to the positive controls MMC and CP were 67% and 50% without or with S9 metabolic activation (supplementary materials Table S5 and S6), respectively. The types of aberrations observed included polyploidy, chromatid gap, break, fragments, deletion, tiny bodies, double centromere, centromere ring, and non-centromere ring (supplementary materials Table S7 and S8). These results indicated a strong chromosomal aberration effect. Furthermore, these results demonstrated that the experimental system was stable, valid, and reliable. Compared to the vehicle group, there were no concentration-related increases in the rates and types of metaphases with aberrant chromosomes at 6 h or 24 h, with or without the S9 mammalian metabolic activation, in the IAAS-exposed group.

#### In Vivo Mammalian Erythrocyte Micronucleus Test

No IAAS-related adverse reactions of mice, including changes in general appearances or body weight, were observed throughout the trial period. The positive control CP increased the frequency of MNPCEs to 13.7‰ compared with that in the vehicle group (1‰, p < 0.01; Supplementary Table S9). This result indicated that the experimental conditions used in this study were acceptable. Oral administration of 200, 400, and 800 mg/kg bw doses of IAAS for 7 days did not result in increased frequencies of MNPCEs or ratios of PCE/(PCE + NCE) compared with those in the vehicle group (p > 0.05). The PCE/(PCE + NCE) ratio in 400 mg/kg bw group was slightly lower than that in the vehicle group and in the 200 mg/kg bw group (p < 0.01). Because this effect was not dose-dependent, it was not considered biologically significant.

### Safety Pharmacology Studies

#### Assessment of Central Nervous System in Mice

No significant differences were observed prior to dosing or 2 h after a single dose of IAAS at 240, 480, or 960 mg/kg bw compared with the vehicle group. The parameters evaluated were fur and skin appearance, unusual behavior, posture, gait, breath, pupil size, lacrimation, and eye discharge (supplementary materials Table S10).

The sleep rates of the animals in the IAAS groups (240, 480, and 960 mg/kg bw) were not significantly different from those in the vehicle group (p > 0.05). This provided further evidence that IAAS from *A. scholaris* did not exhibit a significant synergistic effect with pentobarbital sodium (supplementary materials Table S11).

The standing times of mice treated with three doses of IAAS were approximately equal to those in the vehicle group (p > 0.05). Although the amount of spontaneous activity in the 960 mg/kg bw group decreased slightly, this difference was not statistically significant (p > 0.05). The results showed that IAAS did not significantly affect locomotor coordination in mice. In contrast, the positive control chlorpromazine hydrochloride significantly reduced the amount of spontaneous activity (p < 0.01, supplementary materials Table S12).

Compared with the vehicle group (71.8 ± 35.0 s, supplementary materials Table S13), none of the animals in the IAAS treatment group showed differences in time on the rotating rod (p > 0.05; 240 mg/kg bw, 94.1 ± 54.0 s; 480 mg/kg bw, 75.3 ± 56.3 s; 960 mg/kg bw, 70.3 ± 40.0 s), which indicated that IAAS did not affect coordination of the mice.

These results indicated that IAAS did not significantly affect the central nervous systems of mice.

#### Assessment of Cardiovascular and Respiratory Systems in Dogs

Anesthetized beagle dogs received one intraduodenal dose of IAAS (10, 30, or 60 mg/kg bw, which represented 5-, 15-, and 30-times the recommended dosages in humans). Blood pressure, heart rate, electrocardiograms, and respiratory parameters were recorded from 30 min pre-dose through 240 min post-dose. The results are summarized in supplementary materials Table S14. Respiratory parameters and blood pressure fluctuated with time and animal anesthesia, which is common following animal surgery [44]. Prior to administration, there was a difference in heart rate between the 30 mg/kg bw dose group (166 ± 41) and the vehicle group (211 ± 8, p < 0.05). Heart rate was significantly lower (p < 0.05) in the vehicle group at 240 min (178 ± 29) following administration compared with heart rate pre-administration (supplementary materials Table S14**)**. No other statistically significant or biologically relevant differences in heart rate or electrocardiogram parameters (P wave, R wave, T wave, ST segments, P-R interval, QRS duration, and Q-T intervals) were observed between animals treated with 10, 30 and 60 mg/kg bw IAAS and those in the vehicle group (supplementary materials Table S14**)**. Animals treated with intraduodenal IAAS at doses of 10, 30, and 60 mg/kg bw did not show any statistically significant or biologically relevant differences in blood pressure (systolic, diastolic and mean blood pressure), or frequency and depth of respiration, compared with those in the vehicle group prior to dosing or at 30, 60, 90, 120, 150, 180, 210, and 240 min post-dosing (supplementary materials Table S15**)**.

## Discussion

Natural drugs have become increasingly popular in healthcare and other industries. However, toxicity data for natural products is limited because these agents are generally perceived as harmless to the body and are often used for self-therapy without supervision. Leaves of *A. scholaris* have traditionally been used to treat whooping cough in Dai people for thousands of years. Previously, our group showed that IAAS of *A. scholaris* was effective for respiratory management, but we had not previously provided a preclinical safety evaluation. Therefore, we evaluated genotoxicity and safety pharmacology of IAAS with the goal of reducing risk to future clinical trial subjects and post-market IAAS users.

Genotoxicity tests play a decisive role in predicting carcinogenicity and reproductive toxicity because compounds that test positive often exhibit strong genotoxicity and carcinogenicity in humans [45]. Genotoxicity includes gene mutation, chromosomal aberration, and DNA damage. We conducted a standard battery tests, including the in vitro Ames test, the in vitro chromosomal aberration test, and the in vivo MN test to evaluate potential genotoxicity of IAAS.

The Ames test was developed by Ames et al. in the early 1970s and is internationally recognized as the standard for determination of drug-induced genetic changes. Most genotoxic carcinogens in rodents and humans can be detected by the Ames test [46]. The Ames test is commonly used as an initial screening tool for genotoxicity, and is particularly sensitive to detection of point mutation induction activity. Mutation of genes results in deficient DNA repair and greatly enhances the sensitivity of these strains to certain mutagens [47]. *Salmonella typhimurium* strains TA97, TA98, TA100, TA102, and TA1535, which exhibit base pair substitutions and frame shift mutation, are often used to detect point mutations in the Ames test [48]. There were no increases in the numbers of revertant colonies in the five *S. typhimurium* strains tested at any concentration (31.25, 62.5, 125, 250, or 500 μg/plate) of IAAS, regardless of metabolic activation induction. This study showed that IAAS was not mutagenic toward these five stains of *S. typhimurium*.

The in vitro chromosomal aberration test is used to identify structural chromosomal aberrations, which are classical genotoxic markers of tumor initiation and development [49]. Chinese hamster lung cells were chosen as the detection system because they are sensitive to mutagenic agents, fewer chromosomes allows for simplified observation and scoring, and they are relatively easy to obtain, grow, and maintain [50]. We observed no significant increases in the number of metaphases with structural aberrations following treatment with 710 μg/mL of IAAS 6 h with metabolic activation or 24 h without metabolic activation. These results demonstrated that IAAS did not induce clastogenicity in this study.

The genotoxic potential of IAAS is an important parameter to evaluate because this extract is used by humans. Therefore, next we examined whether treatment with IAAS resulted in chromosome damage using the MN test. This test is used to identify agents that cause chromosome breaks and aneuploidy inducers, since one or more chromosomes in anaphase of cell division can also form micronuclei [51]. Increased frequency of MNPCEs indicates chromosomal damage and cancer risk [52, 53]. In the present study, there was no significant dose-related increase in the number of MNPCEs in response to IAAS treatment at any level. Furthermore, all animals exhibited normal general appearance and body weight did not change in response to IAAS. In addition to genotoxicity, the MN test also allows for evaluation of cytotoxicity through calculation of the PCE/(PCE + NCE) ratio in bone marrow cells. This ratio in mice treated with IAAS did not differ from that in the vehicle group. This ratio was slightly decreased in 400 mg/kg bw group compared to that in the vehicle and 200 mg/kg bw groups, but the absence of dose-dependence indicated that these effects were not IAAS-related.

Safety pharmacology, an important component of preclinical safety evaluation of new drugs, is characterized by investigation of potential adverse physiological effects in response to a test article at exposure levels at or above the therapeutic dose [54]. Safety pharmacology evaluation is divided into two grades according to groups of organ systems: Grade I is a core battery of organ systems, including the central nervous system, cardiovascular system and respiratory system; and Grade II is other organ systems, including the kidney, gastrointestinal tract, and autonomic nervous system. To evaluate the safety pharmacology of IAAS of *A. scholaris*, we evaluated the effects of IAAS on the central nervous, cardiovascular, and respiratory systems of mice and beagle dogs.

A full functional battery of nonclinical safety studies was performed to provide a more in-depth assessment of the ability of IAAS to induce neurotoxicity, including a general behavior test, evaluation of synergistic effects with a subthreshold dose of pentobarbital sodium, the locomotor activity test, and the locomotor coordination test. We administered IAAS orally to mice at doses of 240, 480, and 960 mg/kg bw, which were well above the therapeutic dose in humans of 2 mg/kg bw [55] (120-, 240-, and 480-times the recommended human dose). The results showed an increasing trend in amounts of sleeping animals at 960 mg/kg bw when compared to the vehicle group. However, these effects were not considered IAAS-related side effects because other supportive observations associated with autonomic nervous defects such as urine pools, piloerection, lacrimation, salivation, and papillary reflex were not observed in this group or in the vehicle group. The results of these studies indicated that a single oral dose of IAAS at 240, 480, 960 mg/kg bw did not induce toxicity in the central nervous system. The absence of neuropathy in mice given IAAS was consistent with results of previous animal toxicology studies that demonstrated greater safety margins for IAAS [62].

Twenty-four anesthetized beagle dogs were used to assess potential cardiovascular and respiratory system side effects of a single intraduodenal dose of IAAS. There were no effects on blood pressure (systolic, diastolic and mean blood pressure), heart rate, or electrocardiogram parameters (P wave, R wave, T wave, ST segments, P-R interval, QRS duration, and Q-T intervals) in any of the IAAS treated dogs. These results were consistent with the results of a previous 13-week chronic toxicity study in dogs that demonstrated greater safety margins for IAAS (data not published). Based on the results of this study, 10, 30, and 60 mg/kg bw intraduodenal doses of IAAS did not induce changes in blood pressure, heart rate, or electrocardiogram parameters at 0, 30, 60, 90, 120, 150, 180, 210, or 240 min post-dosing in beagle dogs.

Indole alkaloids extract of *A. Scholaris* leaves, an extract rich in indole alkaloids, did not induce genotoxicity or adverse effects in the central nervous system, cardiovascular system, or respiratory system.

## Conclusions

In conclusion, genotoxic effects of IAAS were not observed in our studies according to the Ames test, the chromosomal aberration assay, and the MN test, and any adverse effects in any of the major vital organ systems were not induced, as determined using the safety pharmacology core battery. These results met the requirements for regulatory safety submission as defined by the CFDA, and supported a clinical trial application. These findings may provide a better understanding of any adverse events observed during a clinical trial.

## Materials and Methods

### Plant Material

Leaves of *A. scholaris* were purchased from Datang-Hanfang Medicine Co. Ltd. (Pu’er, China). Plants were collected in 2006 in Pu’er city of Yunnan Province, People’s Republic of China, and identified by Dr. Xiao-Dong Luo, Kunming Institute of Botany, Chinese Academy of Sciences. Plant names were checked using the website https://www.theplantlist.org. A voucher specimen (No. Luo20060407) was deposited in State Key Laboratory of Phytochemistry and Plant Resources in West China, Chinese Academy of Sciences.

### Alkaloid Preparation

Dried and powdered leaves of *A. scholaris* were extracted by refluxing in 90% EtOH (four times, 3 h each), and the solvent was evaporated under vacuum to obtain the ethanolic extract. The ethanolic extract was dissolved in 0.3% aqueous HCl solution, filtered, and the residue was used as the non-alkaloid fraction. The acidic solution was adjusted to pH 9–10 using 10% aqueous ammonia, then extracted using EtOAc to obtain the IAAS fraction (batch number, 20,070,512). High performance liquid chromatography with ultraviolet/visible detection was used to determine the presence of the four major alkaloids of IAAS (Supplementary materials, S1.1.–1.2.). Representative HPLC/UV chromatograms of IAAS are presented in Fig. S1B. The chromatographic profile showed four peaks with retention times of 7.464 (19-epischolaricine), 7.965 (scholaricine), 12.810 (vallesamine), and 21.874 (picrinine) min.

### Chemicals

All chemical reagents, solvents, pharmaceuticals, and other chemicals used in this study were of analytical or pharmaceutical grade. Agar was purchased from Shanghai Shanpu Chemical Co., Ltd. (Shanghai, China). Peptone was sourced from Oxoid Ltd. (Hampshire, England). 1640 medium was purchased from GIBCO (Massachusetts, USA). 2-aminofluorene (2-AF) was purchased from Merck Schuchardt OHG (Massachusetts, USA). Sodium azide (NaN_3_) was purchased from Sangon Biotech Co., Ltd. (Shanghai, China). Phenobarbital was provided by Panya Chemical Co. Ltd (Guangzhou, China) Beta-naphthoflavone was purchased from Alfa Aesar (Aksaray, Turkey). Beta-nicotinamide adenine dinucleotide phosphate monosodium salt (NADP) was purchased from Amresco (Ohio, USA). Pentobarbital sodium and dextrose-6-sodium phosphate (G-6-P) was purchased from Sigma-Aldrich Co. (Saint Louis, USA) Cyclophosphamide (CP) was purchased from Heng Rui Pharmaceuticals (Jiangsu, China). Colchicine was purchased from Beijing Xinjingke Biotechnologies Co., Ltd. (Beijing, China). Mitomycin C (MMC) was purchased from Merck (New York, USA).

### Experimental Animals

Specific pathogen-free Institute of Cancer Research (ICR) mice (18–20 g) were purchased from Kunming Medical University, Kunming City, Yunnan Province, People’s Republic of China (license no. SCXK K2005-0008). The animals were housed in a room maintained at 24 ± 1 °C and 50%–60% relative humidity with a 12 h light–dark cycle, with the lights on from 9:00 to 21:00. The animals were acclimatized to the laboratory environment for 7 days and allowed free access to water and a standard diet prior to initiation of treatment.

Twelve male and 12 female purebred beagle dogs aged 6–10 months (body weights 7–10 kg) in good health were provided by Guangzhou Pharmaceutical Industry Research Institute (Guangzhou, China). All dogs were inoculated with the relevant vaccines and tested for parasites, and hematological and serum biochemical parameters prior to arrival at our laboratory. The dogs were housed (one per stainless steel cage) in a room with a temperature range of 21–26 °C and relative humidity of 40–70% with a 12 h light–dark cycle and air ventilation rated of 9 changes/hour. Food and water were freely available. Three or four weeks of quarantine were required prior to IAAS administration.

Our experiments were reviewed and approved by the Institutional Animal Care and Use Committee of the Yunnan Institute of Medical Material. All animals were fasted but had access to water for 10–12 h prior to initiation of the experiment. The study was conducted in accordance with the Animal Welfare Act.

### Genotoxicity Test

#### Bacterial Reverse Mutation Test

The bacterial reverse mutation test was performed according to the CFDA Guideline for the Testing of Genotoxicity of Drugs No.[ZH] GPT2-1 [56] and the OECD Guideline for Testing of Chemicals No. 471 [57] using the plate incorporation method, as described by Ames et al. [48] and the laboratory's standard operating procedures (SOPs), using *Salmonella typhimurium* (*S. typhimurium*) strains TA97, TA98, TA100, TA102, TA1535 gifted by the National Beijing Center for Drug Safety Evaluation and Research (Beijing, China). The following technique was performed for each of the five *S. typhimurium* strains (TA1535, TA97, TA98, TA100 and TA102) with or without metabolic activation of IAAS by the S9 rat liver homogenate fraction. Briefly, 0.1 mL of a bacterial suspension from a culture agitated overnight at 37 °C, with or without the addition of 0.5 mL of the rat S9 mix metabolic activation system, was incubated with 0.1 mL of IAAS suspended in 1.7% Tween-80/sterile water for injection. The final concentration of IAAS ranged from 31.25 to 500 μg/plate. The mixture was then added to 2.5 mL of top agar containing 10% 0.5 mM biotin histidine solution kept in a state of superfusion at 45 °C. Then, the mixture was stirred using a vortex oscillator and being spread onto petri dish preloaded with 20 mL of minimal agar. Three plates were used per concentration. The plates were then incubated for approximately 48 h at 37 °C. After the 48-h incubation period, colonies of revertants were counted in each plate. Solvent controls (0.1 mL 1.7% Tween-80 solvent/plate) were evaluated under the same conditions. The positive control without metabolic activation was NaN_3_ (1.5 μg/plate) for TA100 and TA1535, and the positive control with metabolic activation was 2-AF (20 μg/plate) for all *S. typhimurium* strains. The highest analyzable IAAS concentration for mutagenicity determination without a bacterial toxicity response was 500 μg/plate in the absence or presence of metabolic activation, according to pilot test. A positive response was defined as an at least twofold (TA98, TA100, and TA102) or threefold (TA1535 and TA97) increase in the mean number of revertants/plate, and demonstrated concentration-dependence in response to the test substance. The results are expressed as the mean number of revertants from six plates at each IAAS concentration.[58].

The S9 rat liver homogenate was prepared by combining phenobarbital with β-naphthoflavone in male rats via intraperitoneal injection, as previously described [48]. The components of the S9 fraction (per 100 mL) included 90 mL of S9 cofactor (0.162 g MgCl_2_·6H_2_O, 0.25 g KCl, 3.58 g NaH_2_PO_4_·12H_2_O, 1.36 g KH_2_PO_4_, 153 mg G-6-P, and 306 mg NADP) and 10 mL of S9 homogenate.

#### In Vitro Mammalian Chromosomal Aberration Test

The clastogenic potential of IAAS was assessed according to the CFDA Guideline for the Testing of Genotoxicity of Drugs No.[ZH] GPT2-1 [56] and the OECD Guideline for Testing of Chemicals No. 473 [59], and in accordance with procedures established by Brusick [60] and our laboratory's SOPs.

Chinese hamster lung (CHL) cells were obtained from Conservation Genetics Kunming Cell Bank, Chinese Academy of Sciences (Yunnan, China), and cultured in supplemented 1640 medium at 37 °C in a humidified 5% CO_2_ atmosphere. The half inhibitory concentration (IC_50_) of IAAS against CHL cells was determined using trypan blue staining assay. Two independent experiments (experiments A and B) were conducted in triplicate at IC_50_, 1/2 IC_50_, 1/4 IC_50_, 1/8 IC_50_, and 1/16 IC_50_ (mg/mL). IAAS was suspended in 1.7%Tween-80/sterile water for injection. In experiment A (with metabolic activation), CHL cultures (5 × 10^3^ cells/bottle) were exposed to vehicle control or each test article concentration for a 6-h period with S9 metabolic activation. Groups of cells were also exposed to the positive control (CP, 20 μg/mL) with S9 metabolic activation. Experiment B differed from experiment A in that cells were exposed to IAAS treatment for 24 h without S9 metabolic activation. The positive control for experiment B without metabolic activation was MMC (2.5 μg/mL). Vehicle control (0.1 mL 1.7% Tween-80 solvent/bottle) was evaluated under the same conditions.

Cells were treated with colchicine (1 μg/mL) for 4 h prior to harvesting. Sampling was performed 24 h after the start of treatment. Following the selection period, the cells were swollen using 0.075 M hypotonic KCl solution for 10 min at room temperature. Then, 5 mL of fixative (methanol:glacial acetic acid; 3:1, v/v) was added, and the cell suspension was refrigerated for 20 min at 4 °C. The suspension was then centrifuged at 1,500 rpm for 5 min. Two slides were prepared from each suspension of fixed cells, and the slides were air-dried and stained with 3% Giemsa solution for determination of chromosomal aberration frequencies. The slides were evaluated in 100 well-spread metaphases for each concentration using a double-blinded procedure. Chromosome number anomalies and structural aberrations were the main types of aberrations observed in this study. Structural aberrations included polyploidy, chromatid gap, break, fragments, deletion, tiny bodies, double centromere, centromere ring, and non-centromere ring.

#### In Vivo Mammalian Erythrocyte Micronucleus Test

The study was designed in accordance with the CFDA Guideline for the Testing of Genotoxicity of Drugs No. [ZH] GPT2-1 [56] and the OECD Guideline for Testing of Chemicals No. 474 [61]. Mice were randomly divided into five groups, including three dosing groups, a vehicle group, and a positive control group. Each group contained ten animals. The indole alkaloids extract was suspended in 1% carboxymethylcellulose sodium (CMC-Na) to prepare doses of 200, 400, and 800 mg/kg bw. The formulations were prepared fresh on the day of dosing and used within 2 h. This dose was determined from acute toxicity tests on mice in which oral administration of IAAS (3.1 g/kg bw) resulted in a 10% death rate [62].

The IAAS and vehicle control groups were treated by gavage for 7 consecutive days, and the positive control (CP dissolved in normal saline, 40 mg/kg bw) was treated once via intraperitoneal injection. All volumes were 20 mL/kg bw. The mice were examined for visible signs of reactions to the treatments following administration. Body weight was measured on the day in which the animals were placed in groups and on the days in which the animals were dosed. The animals were sacrificed 24 h after the last dose. A small amount of bone marrow extruded from the sternal stems of the animals was mixed with fetal bovine serum, smeared uniformly on standard microscope slides, and dried at room temperature. The slides were fixed for a minimum of 5 min in methanol, then stained with Giemsa solution (Leagene Biotechnology Co., Ltd., Beijing, China). A total of 2000 polychromatic erythrocytes (PCEs) per animal were assessed for the frequency of micronucleated polychromatic erythrocytes (MNPCEs). The proportion of PCEs to normochromatic erythrocytes (NCE) [PCE/(PCE + NCE) ratio] was calculated by counting 200 immature erythrocytes.

### Safety Pharmacology Studies

Safety pharmacology assays for *A. scholaris* were conducted in mice and dogs according to the CFDA Guideline for the Testing of Safety Pharmacology Studies of Drugs No.[Z] GPT1-1 [63] and ICH S7A guideline for safety pharmacology studies for human pharmaceuticals [64].

#### Assessment of Central Nervous System in Mice

##### General Behavior Test

Twenty female and 20 male ICR mice were allocated to 4 groups, each containing 5 females and 5 males. The groups include a vehicle group (1% CMC-Na, 20 mL/kg bw) and IAAS (240, 480, 960 mg/kg bw). Doses were determined using the effective dose (30 mg/kg bw) and the half lethal dose (2.2 g/kg bw) in mice [62]. The animals were observed for animal moving around in cage, body position (observation in the open arena), ataxic gait, respiration (observation in arena for 10 s), muscle tone (observation in the open-arena for 1 min), skin color, pupil dilation (held gently vertically and then moved slowly towards light source), lachrymation and spontaneous locomotor activity for 2 h after a single dose.

##### Synergistic Effect of IAAS With a Subthreshold Dose of Pentobarbital Sodium

Twenty-five female and 25 male mice were divided into 5 groups, each containing 5 female and 5 male. These groups included the vehicle group (1% CMC-Na, 20 mL/kg bw), the positive control group (diazepam, 2.5 mg/kg bw), and IAAS groups (240, 480, 960 mg/kg bw). Thirty minutes after single oral administration, the mice were injected intraperitoneally with a sub-hypnotic dose of pentobarbital sodium (24 mg/kg bw, 10 mL/kg). The induction dose of pentobarbital sodium was based on the results of preliminary studies, which showed that the sleep rates in the vehicle group in response to 28 mg/kg bw and 24 mg/kg bw pentobarbital were 70% and 20%, respectively (Data not shown). The number of animals that went to sleep within 10 min was recorded, and the percentage of sleep onset was calculated.

##### Locomotor Activity Test

Twenty-five female and 25 male mice were divided into 5 groups, each containing 5 female and 5 male. These groups included a vehicle group (1% CMC-Na, 20 mL/kg bw), a positive control (chlorpromazine hydrochloride, 10 mg/kg bw), and IAAS groups (240, 480, 960 mg/kg bw). Spontaneous activity was detected using an autonomous activity instrument (ZIL-2 locomotor activity tester; Chinese Academy of Medical Sciences, China). The mice were placed individually in activity cages individually 30 min after a single oral dose of IAAS, then allowed to acclimate to the environment for 3 min. Then, horizontal locomotor activity was measured and recorded automatically for 5 min using a micro-computer control system.

##### Locomotor Coordination Test

Any animal that stayed on the rotating rod at a constant speed of 30 rad/min for less than 15 s was regarded as unqualified. Qualified animals were placed in groups that received the same treatments as those in the”General behavior test” section. The standing time of the mice was detected using a DXP-2 RotaRod System instrument (Chinese Academy of Medical Sciences, China) at a speed of 30 rpm. The mice were placed on the rotating rod individually at 30 min after a single oral dose of IAAS, and each animal was subjected to the test in triplicate. The mean values of the three trials was calculated. Residence time longer than 180 s was recorded as 180 s.

#### Assessment of Canine Cardiovascular and Respiratory Systems

Based on our previous studies, the IAAS effective dose for improving airway inflammation in rats was 15 mg/kg bw [35]. Using a body surface area conversion, the IAAS effective dose for dogs was approximately 4.5 mg/kg bw. In this experiment, 2.2-, 6.6-, and 13.2-fold the effective dose of IAAS was used, and defined as the 10, 30, and 60 mg/kg bw treatment groups, respectively. We administered IAAS intraduodenally. Each of the 4 groups (vehicle and the IAAS groups) contained 3 male and 3 female dogs.

The dogs were anesthetized with an intravenous injection of pentobarbital sodium (30 mg/kg bw). Temperature was maintained in the range of 36–38 °C. Electrocardiogram (ECG) leads were affixed to limbs to determine ECG II, and a respiratory transducer probe was placed in the nasal cavity to measure breathing depth and frequency. A heparin-filled (600 U/mL) catheter was inserted into the femoral artery and connected to a pressure sensor to measure blood pressure. Following surgery, ECG signal recordings including P wave, R wave, T wave, ST segments, P-R interval, QRS duration, and Q-T intervals, and heart rate, were measured using a RM6240C physiological signal collection system (Beijing Jinyang Wanda Technology Co. Ltd, Beijing, China). Respiratory functions such as respiratory frequency and depth were also monitored using this instrument. Blood pressure, which included systolic pressure, diastolic pressure, and mean arterial pressures, was measured in mmHg.

Each of these parameters was measured at 30 min prior to administration of IAAS. Following collection of a stable baseline, 10, 30, or 60 mg/kg bw of IAAS was administered over 30 min via the duodenum, and each parameter was recorded at 30, 60, 90, 120, 150, 180, 210, and 240 min post-administration. Dosing was performed at a volume of 1 mL/kg bw. Animals received 1% CMC-Na solution as the vehicle control. All dogs were monitored throughout the experiment to assess signal quality, and to monitor clinical signs and general health.

### Statistical Analysis

#### Bacterial Reverse Mutation Test

Data were presented means ± standard deviations (SD) to summarize manual counting of the colonies. The historical control database maintained by our laboratory was considered to determine whether colonies should be counted. The result was judged as positive when one of the below listed criteria had been met: (1) At least one of the test doses and the positive control induced a biologically significant increase (twofold or threefold increase in the mean number TA97, TA98, TA100, TA102, and TA1535 strains) compared with the concurrent negative control; and (2) dose-dependent increases were observed.

#### In Vitro Mammalian Chromosomal Aberration Test

The judgment criteria are presented as follows: aberration rate < 5%, (−); < 10% (±); > 10% (+); > 20% (++); > 50% (+++).

#### In Vivo Mammalian Erythrocyte Micronucleus Test and Safety Pharmacology Assay

Data are presented as means ± SD. Statistical evaluation was performed using SPSS 12.0 software (Chicago, IL, USA) and variations in data for all parameters were assessed for homogeneity using Bartlett’s procedure. In cases where data were homogeneous, one-way analysis of variance for homogeneity (ANOVA) was used. In heterogeneous cases, the Kruskal–Wallis test was used. When significant differences were indicated, LSD and Dunnett’s T3 multiple tests were used to compare the control and treated groups. The nonparametric test was used to analyze ranked data. Results were considered significant at *p* values < 0.05. The Fisher's exact and chi-square tests were used to analyze the number of animals that fell asleep.

## Electronic supplementary material

Below is the link to the electronic supplementary material.

Supplementary file1 (PDF 1175 kb)

## Abbreviations

Ames test: Bacterial reverse mutation test
CFDA: China Food and Drug Administration
CHL: Chinese hamster lung
CMC-Na: Carboxymethylcellulose sodium
CP: Cyclophosphamide
ECG: Leads of electrocardiogram
G-6-P: Dextrose-6-sodium phosphate
IAAS: Indole alkaloids extract
IC_50_: Half inhibitory concentration
MMC: Mitomycin C
MNPCEs: Micronucleated polychromatic erythrocytes
MN test: Mammalian erythrocyte micronucleus test
NADP: β-Nicotinamide adenine dinucleotide phosphate monosodium salt;
NaN_3_: Sodium azide
NCE: Normochromatic erythrocytes
OECD: Organization for Economic Co-operation and Development
PCEs: Polychromatic erythrocytes
2-AF: 2-Aminofluorene

## References

[1] Li PT, Leeuwenberg AJM, Middleton DJ (1995). Ann. Mo. Bot. Garden.

[2] Zhang WH, Liu Y, Wang XQ, Fang P, Song HY, Wang J, Shan TJ (2017). Chin. J. Trop. Agric..

[3] Singh MP, Panda H (2005). Medicinal Herbs with Their Formulations.

[4] Cummings J (2014). Tech. Eng..

[5] Vedavathy S, Sudhakar A, Mrdula V (1997). Anc. Sci. Life.

[6] Sharma H, Kumar A (2011). J. Med. Plants Res..

[7] Sen S, Chakraborty R, De B, Devanna N (2011). J. For. Res..

[8] Editorial Board of Chinese Flora, Chinese Academy of Sciences (1977). Flora of China.

[9] Jaipuriar MK (2007). Herbs of Tribal Land Jharkhand.

[10] Saikia B (2006). Indian J. Tradit. Knowl..

[11] Ayyanar M, Ignacimuthu S (2005). J. Ethnopharmacol..

[12] Compiling Group of Yunnan Traditional Chinese Medicine. Yunnan People's Press, Kunming (1977)

[13] Cai XH, Shang JH, Feng T, Luo XD (2010). Z. Nat..

[14] Chen YY, Yang J, Yang XW, Khan A, Liu L, Wang B, Zhao YL, Liu YP, Ding ZT, Luo XD (2016). Tetrahedron Lett..

[15] Yang XW, Song CW, Zhang Y, Khan A, Jiang LP, Chen YB, Liu YP, Luo XD (2015). Tetrahedron Lett..

[16] Yang XW, Qin XJ, Zhao YL, Lunga PK, Li XN, Jiang SZ, Cheng GG, Liu YP, Luo XD (2014). Tetrahedron Lett..

[17] Yang XW, Luo XD, Lunga PK, Zhao YL, Qin XJ, Chen YY, Liu L, Li XN, Liu YP (2015). Tetrahedron.

[18] Qin XJ, Zhao YL, Lunga PK, Yang XW, Song CW, Cheng GG, Liu L, Chen YY, Liu YP, Luo XD (2015). Tetrahedron.

[19] Feng T, Cai XH, Zhao PJ, Du ZZ, Li WQ, Luo XD (2009). Planta Med..

[20] Yang XW, Yang CP, Jiang LP, Qin XJ, Liu YP, Shen QS, Chen YB, Luo XD (2014). Org. Lett..

[21] Pan ZQ, Qin XJ, Liu YP, Wu T, Luo XD, Xia CF (2016). Org. Lett..

[22] Cai XH, Du ZZ, Luo XD (2007). Org. Lett..

[23] Cai XH, Tan QG, Liu YP, Feng T, Du ZZ, Li WQ, Luo XD (2008). Org. Lett..

[24] Qin XJ, Zhao YL, Song CW, Wang B, Chen YY, Liu L, Li Q, Li D, Liu YP, Luo XD (2015). Nat. Prod. Bioprospect..

[25] Zhang ZY, Luo XD, Li S (2014). J. Med. Plants Res..

[26] Zhou H, He HP, Luo XD, Wang YH, Yang XW, Di YT, Hao XJ (2005). Helv. Chim. Acta..

[27] Feng T, Cai XH, Du ZZ, Luo XD (2008). Helv. Chim. Acta..

[28] Liu L, Chen YY, Qin XJ, Wang B, Jin Q, Liu YP, Luo XD (2015). Fitoterapia.

[29] Xu Y, Feng T, Cai XH, Luo XD (2009). Chin. J. Nat. Med..

[30] Du GS, Cai XH, Shang JH, Luo XD (2007). Chin. J. Nat. Med..

[31] Cai XH, Liu YP, Feng T, Luo XD (2008). Chin. J. Nat. Med..

[32] Du GS, Shang JH, Cai XH, Luo XD (2007). Acta Bot. Yunnanica..

[33] Shang JH, Cai XH, Feng T, Zhao YL, Wang JK, Zhang LY, Yan M, Luo XD (2010). J. Ethnopharmacol..

[34] Shang JH, Cai XH, Zhao YL, Feng T, Luo XD (2010). J. Ethnopharmacol..

[35] Zhao YL, Shang JH, Pu SB, Wang HS, Wang B, Liu L, Liu YP, Mei SH, Luo XD (2016). J. Ethnopharmacol..

[36] Zhao YL, Cao J, Shang JH, Liu YP, Khan A, Wang HS, Qian Y, Liu L, Ye M, Luo XD (2017). Phytomedicine.

[37] Zhao YL, Yang ZF, Shang JH, Huang WY, Wang B, Wei X, Khan A, Yuan ZW, Liu YP, Wang YF, Wang XH, Luo XD (2018). J. Ethnopharmacol..

[38] Hou YY, Cao XL, Dong LY, Wang LQ, Cheng BF, Shi Q, Luo XD, Bai G (2012). J. Chromatogr. A..

[39] Hou YY, Cao XL, Wang LQ, Cheng BF, Dong LY, Luo XD, Bai G, Gao WY (2012). J. Chromatogr. B..

[40] Cao J, Shen HM, Wang Q, Qian Y, Guo HC, Li K, Qiao X, Guo DA, Luo XD, Ye M (2015). J. Chromatogr. B.

[41] Baliga MS, Jagetia GC, Ulloor JN, Baliga MP, Venkatesh P, Reddy R, Rao KV, Baliga BS, Devi S, Raju SK, Veeresh V, Reddy TK, Bairy KL (2004). Toxicol. Lett..

[42] Jagetia GC, Baliga MS (2003). Birth Defects Res. B.

[43] CFDA (2003). Good Laboratory Practice Regulations.

[44] Runcie MJ, Ulman LG, Potter EK (1995). Regul. Pept..

[45] Galloway SM (1994). Environ. Mol. Mutagen..

[46] Shin KY, Won BY, Ha HJ, Yun YS, Lee HG (2017). Regul. Toxicol. Pharmacol..

[47] Choi JS, Cheon EJ, Kim TU, Moon WS, Kim JW, Kim MR (2014). Biol. Pharm. Bull..

[48] Ames BN, McCann J, Yamasaki E (1975). Mutat. Res..

[49] Matsushima T, Hayashi M, Matsuoka A, Ishidate M, Miura KF, Shimizu H, Suzuki Y, Morimoto K, Ogura H, Mure K (1999). Mutagenesis.

[50] Koyama H, Yatabe I, Ono T (1970). Exp. Cell Res..

[51] Ashby J (1985). Mutat. Res..

[52] Suárez S, Sueiro RA, Araujo M, Pardo F, Alvarez A (2007). Mutat. Res..

[53] Fenech M (2000). Mutat. Res..

[54] ICH, S7A Safety Pharmacology Studies for Human Pharmaceuticals (2013)

[55] Li Rui, Zi Ming-Jie, Gou Zhong-Ping, Zhao Yun-Li, Zhang Wan-Tong, Lu Fang, Cao Wei-Yi, Zhao Ying-Pan, Li Qing-Na, Zhao Yang, Wang Shu-Ge, Gao Hong-Yang, Sun Ming-Yue, Luo Xiao-Dong, Xiong Zhi-Li, Gao Rui (2019). Pharmacokinetics and safety evaluation in healthy Chinese volunteers of alkaloids from leaf of Alstonia scholaris: A multiple doses phase I clinical trial. Phytomedicine.

[56] CFDA, Guideline for the testing of genotoxicity of drugs, Vol. [ZH] GPT2-1 (2007)

[57] OECD, Guideline for Testing of Chemicals No. 471: Bacterial reverse mutation test (1997)

[58] OECD, Guideline for the Testing of Chemicals No. 471: Bacterial Reverse Mutation Test (1997)

[59] OECD, Guideline for Testing of Chemicals No. 473: *In vitro* mammalian chromosomal aberration test (2016)

[60] Brusick D (1989). Genetic Toxicology.

[61] OECD, Guideline for Testing of Chemicals No. 474: mammalian erythrocyte micronucleus test (2016)

[62] Zhao YL, Su M, Shang JH, Wang X, Njateng GSS, Bao GL, Ma J, Sun QD, Yuan F, Wang JK, Luo XD (2020). Nat. Prod. Bioprospect..

[63] CFDA, Guideline for the testing of safety pharmacology studies of drugs. In 2007; Vol. [Z] GPT1-1

[64] ICH, S7A: Safety Pharmacology Studies for Human Pharmaceuticals (2001) 12356097

