# Supplementary material for: Genotoxicity and Safety Pharmacology Studies of Indole Alkaloids Extract from Leaves of Alstonia scholaris (L.) R. Br
Source: Nat Prod Bioprospect. 2020 Apr 30;10(3):119–29. doi: 10.1007/s13659-020-00242-4 (PMC7253540; doi:10.1007/s13659-020-00242-4)
Supplement: Supplementary file 1 — Supplementary file1 (PDF 1175 kb) [file 13659_2020_242_MOESM1_ESM.pdf]

Supplementary materials for

Genotoxicity and safety pharmacology studies of indole alkaloids extract from leaves of *Alstonia scholaris* (L.) R. Br.

Yun-Li Zhao [a,c,1], Min Su [b,1], Jian-Hua Shang [b,c,1], Xia Wang [b], Guang-Lei Bao [b], Jia Ma [b], Qing-Di Sun [d], Fang Yuan [b], Jing-Kun Wang [b,*], Xiao-Dong Luo [a,c,*]

[a] *State Key Laboratory of Phytochemistry and Plant Resources in West China, Kunming Institute of Botany, Chinese Academy of Sciences, Kunming 650201, P. R. China*

[b] *Yunnan Institute of Medical Material, Kunming 650111, P. R. China*

[c] *Key Laboratory of Medicinal Chemistry for Natural Resource, Ministry of Education and Yunnan Province, School of Chemical Science and Technology, Yunnan University, Kunming 650091, People's Republic of China*

[d] *Jiangsu Nhwa Pharmaceutical Co., Ltd, Xuzhou, 221009, P. R. China*

---

* Corresponding author. Tel.: +86 871 65223177; fax: +86 871 65220227.

E-mail address: xdluo@mail.kib.ac.cn (X.-D. Luo); wjkyimm@163.com (J.-K. Wang)

[1]These authors contributed equally.

Supplementary Text

S1. The “hot off the press” compounds and total synthesized *Alstonia* alkaloids 3

Table S1 Eight “hot off the press” compounds as specified in *Natural Products Report* 3

Table S2 Sixteen total synthesized Alstonia alkaloids by chemists 3

S2. Alkaloids quantitative analysis 4

S2.1. Sample preparation 4

S2.2. HPLC/UV quantitative analysis 4

Fig. S1. HPLC/UV chromatograms of total alkaloids (285 nm) 5

S3. The results of bacterial reverse mutation test 6

Table S3 Effect of IAAS on bacterial reverse mutation assay 6

Table S4 Effect of IAAS on cell proliferation activity of CHL cells 7

Table S5 Effect of chromosome aberrations after the exposure of IAAS without S9 8

Table S6 Effect of chromosome aberrations after the exposure of IAAS with S9 9

Table S7 Effect on type of aberration after the exposure of IAAS without S9.. 10

Table S8 Effect on type of aberration after the exposure of IAAS with S9... 11

Table S9 Mammalian erythrocyte micronucleus test in mice following 7 days oral dose of IAAS 12

Table S10 General behavioral activity observation of mice after the administration of IAAS 13

Table S11 Synergistic effect with pentobarbital sodium after the administration of IAAS 14

Table S12 Effects of oral administration of IAAS on locomotor activity test in mice 15

Table S13 Effects of oral administration of IAAS on coordination ability of mice 16

Table S14 Effect of IAAS on on electrocardiogram in anesthetized dogs 17

Table S15 Effect of IAAS on blood pressure and respiratory parameters in anesthetized dogs............... 18

S1. The “hot off the press” compounds and total synthesized *Alstonia* alkaloids

Table S1 Eight “hot off the press” compounds as specified in *Natural Products Report*

No.	Compounds	No.	Compounds
1	E-alstoscholarines (Cai et al., 2007)	5	alstolactine A (Yang et al., 2014a)
2	Z-alstoscholarines (Cai et al., 2007)	6	alstoscholarisine A (Yang et al., 2014b)
3	scholarisine A (Cai et al., 2008)	7	alstoscholarisines F (Yang et al., 2015)
4	alstrostine A (Cai et al., 2011)	8	alstoscholarisines H (Pan et al., 2016)

Table S2 Sixteen total synthesized Alstonia alkaloids by chemists

No.	Compounds	No.	Compounds
1	scholaricine A (Adams et al., 2012, 2013; Smith and Snyder, 2013; Watanabe et al., 2013),	9	scholaricine K (Wang et al., 2017)
2	E-alstoscholarine (Gerfaud et al., 2011)	10	alstolactine A (Wang et al., 2017)
3	Z-alstoscholarine (Gerfaud et al., 2011)	11	alstoscholaricine B (Mason and Weinreb, 2018)
4	alstoscholaricine A (Moreno et al., 2016; Ren et al., 2016)	12	alstoscholaricine C (Mason and Weinreb, 2018)
5	alstoscholaricine H (Pan et al., 2016)	13	alstoscholaricine D (Mason and Weinreb, 2018)
6	scholaricine G (Higuchi et al., 2015; Umehara et al., 2014; Xu et al., 2015; Yang et al., 2014c)	14	alstoscholaricine E (Mason and Weinreb, 2018)
7	scholaricine K (Wang et al., 2017)	15	picrinine (Smith et al., 2014, 2015)
8	alstolactine A (Wang et al., 2017)	16	strictamine (Liang et al., 2016; Ren et al., 2016)

S2. Alkaloids quantitative analysis

S2.1. Sample preparation

The sample of total alkaloids was pulverized into fine powder. Then 1.0 mg of the total alkaloids was accurately weighed and extracted with 1.0 mL of 50% methanol in ultrasonic water bath for 30 min at 25 °C. The sample solution was filtered through a 0.22 μm membrane before use. A 10 μL-aliquot was injected for UHPLC/UV quantitative analysis.

S2.2. HPLC/UV quantitative analysis

The quantitative analysis was performed on an a Agilent 1290 series HPLC system (Agilent Technologies, Germany) comprised a quaternary pump, an auto-sampler, a column temperature controller and a PDA detector. All samples were separated on an ACQUITY UPLC® CSH column (2.1 × 100 mm, 1.7 μm) equipped with a Waters VanGuard pre-column (5 mm × 2.1 mm I.D., 1.8 μm). The mobile phase consisted of acetonitrile (A) and water containing 0.5% trifluoroacetic acid (v/v, B) at the ratio of 18 : 82. The following linear elution gradient was used: 0-10 min, 4-16% A; 10-15 min, 16-20% A; 15-18 min, 20% A; 18-20min, 20-95% A. The flow rate was 0.20 mL/min. The column temperature was maintained at 45 °C. The sample tray temperature was maintained at 25 °C. The detection wavelength was set from 190 to 600 nm, and the samples were detected at 285 nm in accordance with the maximum absorption of the analytes. Typical HPLC/UV chromatograms of TA are presented in Fig. S1. As shown in Fig. S1B, the chromatography profile displayed four peaks with retention times of 7.464 (19-epischolaricine), 7.965 (scholaricine) 12.810 (vallesamine) and 21.874 minutes (picrinine).

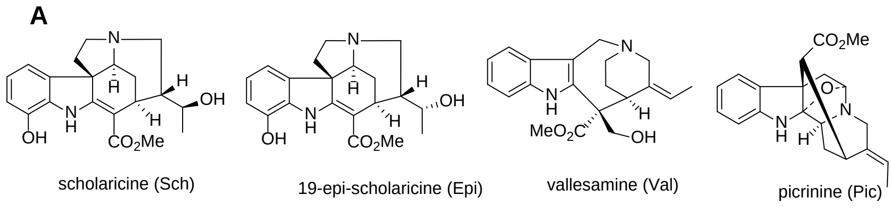

B

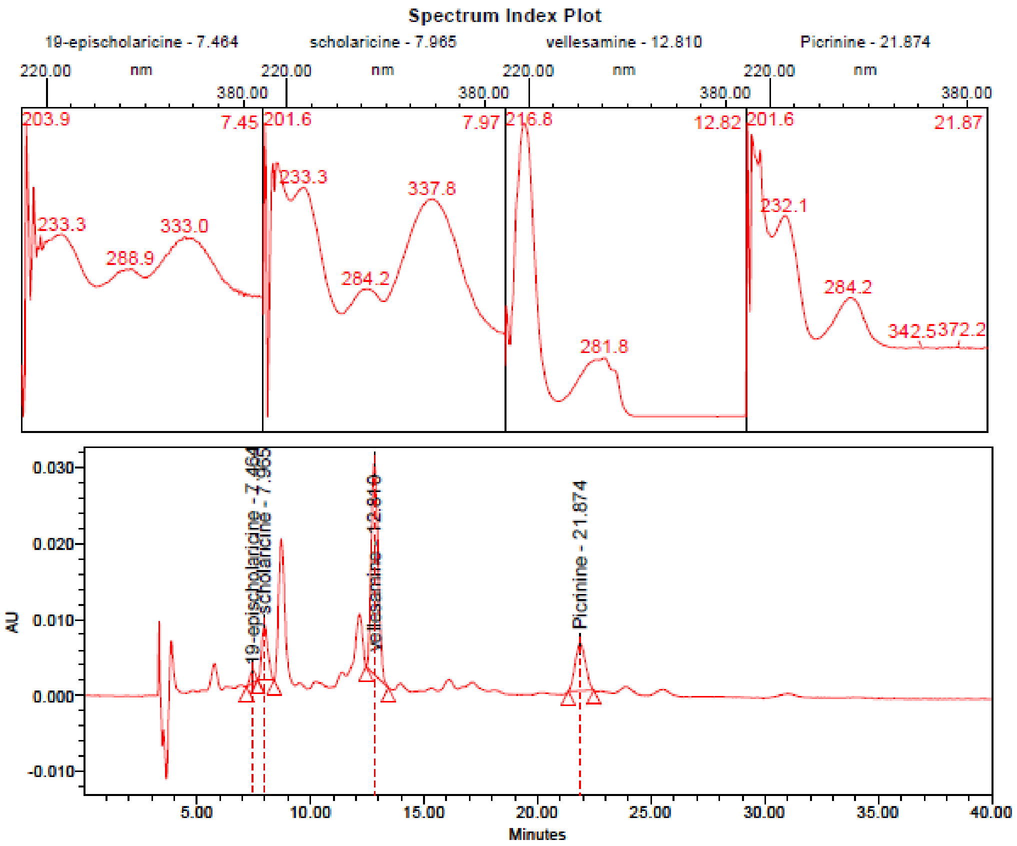

Fig. S1. HPLC/UV chromatograms of total alkaloids (285 nm)

A. Four major alkaloids from leaf of *A. scholaris.*

B. HPLC/UV chromatograms of total alkaloids.

S3. The results of bacterial reverse mutation test

Table S3 Effect of IAAS on bacterial reverse mutation assay

表 6　灯台叶碱提取物药液 Ames 试验结果（个/皿、$\bar{x}$ ±SD）#

组别	剂量（μg/皿）	TA1535		TA97		TA98		TA100		TA102	
		$-S_9$	$+S_9$	$-S_9$	$+S_9$	$-S_9$	$+S_9$	$-S_9$	$+S_9$	$-S_9$	$+S_9$
自发回变	——	18±6	17±6	153±25	148±23	36±8	43±7	129±14	149±11	269±24	286±43
溶媒对照	——	21±6	21±5	123±21	111±14	37±8	45±6	142±37	148±14	260±27	269±26
NaN_3	1.5	462±55**	——	——	——	——	——	712±126**	——	——	——
2-AF	20	——	446±105**	——	842±95**	——	635±137**	——	772±200**	——	683±41**
灯台叶碱提取物	500	21±4	30±10	169±15	162±30	34±7	45±9	138±19	160±33	276±22	271±12
	250	21±6	30±10	165±18	173±16	45±22	49±9	142±14	160±33	251±13	281±64
	125	19±4	18±7	143±17	153±31	45±22	47±10	129±7	156±20	253±11	269±18
	62.5	20±6	19±6	148±25	163±12	42±9	44±8	141±8	146±21	264±24	273±22
	31.25	20±4	14±5	138±16	165±22	46±14	48±12	133±10	161±30	244±10	274±23

统计学分析：# 两次试验平均值 $\bar{x}$ ±SD；** 回变菌落数是自发回变菌落数的 2 倍以上

Notes:

-S9: without metabolic activation; +S9: with metabolic activation

Data were presented means ± standard deviations (SD) after manual counting of the colonies.

** represented the number of colonies was more than 2 fold that of the spontaneous colonies.

Table S4 Effect of IAAS on cell proliferation activity of CHL cells

表 6 灯台叶碱提取物药液对 CHL 细胞存活的影响

组别	浓度（mg/ml）	存活细胞数（$\bar{x}$ ±SD，×10^4 个）	抑制率（%）	IC50（mg/ml）
溶媒对照	——	31.7±9.1	——	——
灯台叶碱提取物	0.3125	18.7±3.8	41.32	
	0.625	14.3±1.8	54.89	
	1.25	10.5±3.0	67.19	0.704
	2.5	10.4±5.7	67.19	
	5.0	6.7±0.3	79.18	

Notes:

The mean ± SD represented the mean value of survival cells after the treatment of IAAS, and the half inhibitory concentration (IC 50) was 0.704 mg/mL.

Table S5 Effect of chromosome aberrations after the exposure of IAAS without S9

表 7　灯台叶碱提取物药液不加 S9 对 CHL 细胞染色体畸变率的影响

组别	浓度（mg/ml）	计数细胞数（个）	畸变细胞数（个）	畸变率（%）	结果判定
空白对照	——	100	1	1.0	（－）
溶媒对照	——	100	1	1.0	（－）
丝裂霉素	0.0125	100	35	67.0	（+++）
灯台叶碱提取物	0.710	100	1	1.0	（—）
	0.355	100	1	1.0	（—）
	0.178	100	0	0.0	（－）
	0.089	100	0	0.0	（－）
	0.044	100	1	1.0	（－）

Notes:

The positive control mitomycin (MMC) was disclosed strong chromosomal aberration effect (+++), and the chromosomal aberration rate was 67.0%.

Table S6 Effect of chromosome aberrations after the exposure of IAAS with S9

表 8　灯台叶碱提取物药液加 S9 对 CHL 细胞染色体畸变率的影响

组别	浓度（mg/ml）	计数细胞数（个）	畸变细胞数（个）	畸变率（%）	结果判定
空白对照	——	100	0	0.0	（－）
溶媒对照	——	100	1	1.0	（－）
环磷酰胺	1.0	100	33	50.0	（++）
灯台叶碱提取物	0.710	100	1	1.0	（—）
	0.355	100	1	1.0	（—）
	0.178	100	1	1.0	（－）
	0.089	100	0	0.0	（－）
	0.044	100	2	2.0	（－）

Notes:

The positive control cyclophosphamide (CP) was disclosed strong chromosomal aberration effect (++), and the chromosomal aberration rate was 50.0%.

Table S7 Effect on type of aberration after the exposure of IAAS without S9

表 9 灯台叶碱提取物药液不加 S9 对 CHL 细胞染色体畸变类型观察结果

组别	浓度（mg/ml）	细胞总数（个）	多倍体	裂隙	断裂	断片	缺失	微小体	双着丝点	着丝点环	无着丝点环	复合射体
空白对照	——	100	1	2	1	0	0	0	0	0	0	0
溶媒对照	——	100	2	1	1	0	0	0	0	0	0	0
丝裂霉素	0.0125	100	1	15	10	7	2	1	3	9	4	31
灯台叶碱提取物	0.710	100	3	3	1	0	0	0	0	0	0	0
	0.355	100	1	1	0	1	0	0	0	0	0	0
	0.178	100	2	2	0	0	0	0	0	0	0	0
	0.089	100	1	2	0	0	0	0	0	0	0	0
	0.044	100	1	1	0	1	0	0	0	0	0	0

Notes:

The positive control mitomycin (MMC) caused CHL cells multiple types of aberration without metabolic activation such as polyploidy, chromatid gap, break, fragments, deletion, tiny bodies, double centromere, centromere ring and non-centromere ring, compound emitter.

Table S8 Effect on type of aberration after the exposure of IAAS with S9

表 10　灯台叶碱提取物药液加 S9 CHL 细胞染色体畸变类型观察结果

组别	浓度（mg/ml）	细胞总数（个）	多倍体	裂隙	断裂	断片	缺失	微小体	双着丝点	着丝点环	无着丝点环	复合射体
空白对照	——	100	1	1	0	0	0	0	0	0	0	0
溶媒对照	——	100	1	1	0	0	1	0	0	0	0	0
环磷酰胺	1.0	100	3	12	4	9	7	0	5	3	1	21
灯台叶碱提取物	0.710	100	1	2	1	0	0	0	0	0	0	0
	0.355	100	0	1	1	0	0	0	0	0	0	0
	0.178	100	1	3	1	0	0	0	0	0	0	0
	0.089	100	0	1	0	0	0	0	0	0	0	0
	0.044	100	1	0	0	1	1	0	0	0	0	0

Notes:

The positive control cyclophosphamide (CP) caused CHL cells multiple types of aberration without metabolic activation such as polyploidy, chromatid gap, break, fragments, deletion, tiny bodies, double centromere, centromere ring and non-centromere ring, compound emitter.

Table S9 Mammalian erythrocyte micronucleus test in mice following 7 days oral dose of IAAS

表 2　灯台叶碱提取物对小鼠体重、微核发生率及 PCE/（PCE＋NCE）比值的影响

组别	剂量（g/kg）	动物数（只）	体重（$\bar{x}$ ±SD，g）		PCE 数（个）	微核数（$\bar{x}$ ±SD）	微核率（$\times10^{-3}$）	PCE/（PCE+NCE）（$\bar{x}$ ±SD）
			给药前	给药第 4d				
溶媒对照	——	10	22.2±1.1	26.5±1.9	20000	1.0±0.3	1.0	0.38±0.08
环磷酰胺	0.040	10	22.2±1.0	26.3±2.6	20000	13.7±6.0**	13.7	0.32±0.10
低剂量	0.2	10	21.8±1.0	26.3±1.1	20000	1.0±0.5	1.2	0.37±0.12
中剂量	0.4	10	21.8±1.1	24.9±1.8	20000	1.1±0.6	1.1	0.27±0.08**▲▲
高剂量	0.8	10	21.9±0.8	25.0±1.8	20000	1.0±0.5	1.0	0.32±0.14

统计学分析：**与溶媒对照组比较有显著性差异（$P<0.01$）；▲▲与低剂量组比较有显著性差异（$P<0.01$）

Notes:

MNPCE: PCE with one or more micronuclei; PCE: Polychromatic erythrocyte; NCE: Normochromatic erythrocyte.

* $p < 0.05$ compared with the vehicle group; ▲▲ $p < 0.01$ compared with the 200 mg/kg.bw group.

Table S10 General behavioral activity observation of mice after the administration of IAAS

表 7　灯台叶碱提取物安全性药理小鼠一般行为活动观察情况

组别	剂量（mg/kg.bw）	动物数（n）	一般行为	精神	姿势	步态	呼吸	瞳孔	分泌物
溶媒对照	-	10	—	—	—	—	—	—	—
灯台叶碱提取物	240	10	—	—	—	—	—	—	—
	480	10	—	—	—	—	—	—	—
	960	10	—	—	—	—	—	—	—

注：一表示正常，+表示异常。

Notes:

No significant changes were observed after single administration of IAAS at doses of 240, 480, 960 mg/kg.bw to mice when compared to the vehicle group prior to dosing and at 2h post dosing, which particularly in fur and skin appearance, unusual behavior, posture, gait, breath, pupil size, lacrimation, eye discharge.

Table S11 Synergistic effect with pentobarbital sodium after the administration of IAAS

表 10 灯台叶碱提取物安全性药理阈下剂量戊巴比妥钠的协同作用

组别	剂量（mg/kg.bw）	动物数（n）	入睡动物数（只）	入睡率（%）
溶媒对照	-	10	3	30
地西泮	2.5	10	9	90**
灯台叶碱提取物	240	10	0	0
	480	10	0	0
	960	10	3	30

统计分析：**示溶媒对照组比较有显著性差异（p <0.01）

Notes:

** $p < 0.01$ compared with the vehicle group

Table S12 Effects of oral administration of IAAS on locomotor activity test in mice

表 12　灯台叶碱提取物安全性药理小鼠自发活动数统计结果（$\bar{x}$ ±SD）

组别	剂量（mg/kg.bw）	动物数（n）	自发活动次数(次)
溶媒对照	-	10	123±52
盐酸氯丙嗪	10	10	34±23**
灯台叶碱提取物	240	10	121±51
	480	10	129±61
	960	10	85±40

Notes:

Data were expressed as mean ± SD.

** $p < 0.01$ compared with the vehicle group.

Table S13 Effects of oral administration of IAAS on coordination ability of mice

表 15　灯台叶碱提取物安全性药理小鼠协调运动统计结果（$\bar{x}$ ±SD）

组别	剂量（mg/kg）	动物数（n）	平均停留时间（sec）
溶媒对照	-	10	71.8±35.0
灯台叶碱提取物	240	10	94.1±54.0
	480	10	75.3±56.3
	960	10	70.3±40.0

Notes:

Data were expressed as mean ± SD.

$p > 0.05$ compared with the vehicle group

Table S14 Effect of IAAS on on electrocardiogram in anesthetized dogs

表 55　灯台叶碱提取物安全性药理心电检测数据统计结果（$\bar{x}$ ±SD）

测定时间	组别	P 波时限 (sec)	P 波电压 (mv)	P-R 间期 (sec)	QRS 间期 (sec)	Q-T 间期 (sec)	S-T 段上抬 (mv)	R 波电压 (mv)	T 波 (mv)	心率（次/分）
给药前 (n=6)	空白对照组	0.08±0.01	0.51±0.07	0.14±0.01	0.06±0.01	0.21±0.02	0.002±0.004	1.04±0.16	0.38±0.09	211±8
	低剂量组	0.08±0.02	0.56±0.09	0.16±0.02	0.05±0.01	0.21±0.03	0.01±0.02	1.20±0.40	0.26±0.10	190±41
	中剂量组	0.08±0.01	0.52±0.12	0.16±0.02	0.06±0.01	0.24±0.05	0.000±0.000	1.71±0.54	0.39±0.07	166±41 △
	高剂量组	0.09±0.01	0.52±0.07	0.15±0.01	0.06±0.00	0.22±0.02	0.000±0.000	1.40±0.72	0.41±0.18	180±13
给药后 30min (n=6)	空白对照组	0.08±0.02	0.49±0.08	0.14±0.01	0.06±0.01	0.20±0.01	0.002±0.004	1.07±0.22	0.36±0.10	194±16
	低剂量组	0.08±0.01	0.56±0.10	0.15±0.02	0.05±0.01	0.21±0.02	0.007±0.010	1.18±0.41	0.27±0.08	180±42
	中剂量组	0.08±0.01	0.53±0.11	0.16±0.02	0.06±0.01	0.24±0.04	0.000±0.000	1.72±0.56	0.38±0.07	169±40
	高剂量组	0.09±0.01	0.52±0.07	0.15±0.01	0.06±0.00	0.21±0.02	0.000±0.000	1.38±0.69	0.42±0.18	179±14
给药后 60min (n=6)	空白对照组	0.08±0.01	0.47±0.08	0.14±0.01	0.06±0.00	0.21±0.009	0.000±0.000	1.05±0.29	0.38±0.10	193±16
	低剂量组	0.07±0.01	0.55±0.09	0.15±0.02	0.05±0.01	0.21±0.02	0.002±0.004	1.11±0.46	0.29±0.06	191±23
	中剂量组	0.08±0.01	0.54±0.11	0.16±0.02	0.06±0.01	0.24±0.04	0.000±0.000	1.71±0.55	0.41±0.10	176±38
	高剂量组	0.09±0.01	0.52±0.07	0.15±0.01	0.06±0.01	0.22±0.02	0.000±0.000	1.38±0.68	0.43±0.18	176±17
给药后 90min (n=6)	空白对照组	0.08±0.01	0.48±0.12	0.15±0.01	0.05±0.01	0.20±0.008	0.000±0.000	1.07±0.32	0.38±0.12	191±17
	低剂量组	0.08±0.01	0.53±0.09	0.15±0.02	0.05±0.01	0.21±0.03	0.002±0.004	0.94±0.62	0.28±0.06	174±46
	中剂量组	0.08±0.005	0.54±0.10	0.16±0.01	0.06±0.01	0.23±0.04	0.000±0.000	1.71±0.55	0.43±0.13	177±37
	高剂量组	0.09±0.01	0.52±0.08	0.15±0.009	0.06±0.01	0.22±0.03	0.000±0.000	1.39±0.68	0.45±0.19	175±21
给药后 120min (n=6)	空白对照组	0.08±0.01	0.50±0.10	0.14±0.01	0.05±0.01	0.20±0.008	0.002±0.004	1.08±0.34	0.39±0.13	187±20
	低剂量组	0.08±0.02	0.53±0.10	0.16±0.02	0.06±0.00	0.22±0.04	0.002±0.004	1.13±0.54	0.38±0.16	176±43
	中剂量组	0.08±0.00	0.55±0.10	0.15±0.01	0.05±0.01	0.22±0.04	0.000±0.000	1.70±0.57	0.45±0.19	180±38
	高剂量组	0.09±0.01	0.52±0.07	0.15±0.01	0.06±0.01	0.21±0.03	0.000±0.000	1.40±0.70	0.48±0.21	173±24

注：药前采集 1 次基础值，*/**与组内比较有显著性差异（p <0.05/0.01），△/△△与同期空白组比较有显著性差异（p <0.05/0.01）。

续表 55

测定时间	组别	P 波时限 (sec)	P 波电压 (mv)	P-R 间期 (sec)	QRS 间期 (sec)	Q-T 间期 (sec)	S-T 段上抬 (mv)	R 波电压 (mv)	T 波 (mv)	心率（次/分）
给药后 150min (n=6)	空白对照组	0.08±0.01	0.50±0.11	0.14±0.00	0.05±0.01	0.21±0.01	0.000±0.000	1.15±0.40	0.40±0.12	186±24
	低剂量组	0.08±0.01	0.50±0.10	0.16±0.02	0.05±0.01	0.22±0.05	0.002±0.004	1.23±0.65	0.34±0.08	174±42
	中剂量组	0.08±0.01	0.54±0.10	0.15±0.01	0.06±0.01	0.22±0.04	0.000±0.000	1.72±0.58	0.44±0.15	179±41
	高剂量组	0.09±0.01	0.52±0.07	0.15±0.01	0.06±0.01	0.21±0.03	0.000±0.000	1.40±0.69	0.48±0.21	175±26
给药后 180min (n=6)	空白对照组	0.07±0.02	0.50±0.10	0.14±0.02	0.05±0.01	0.20±0.01	0.000±0.000	1.20±0.40	0.43±0.14	182±22
	低剂量组	0.07±0.01	0.50±0.10	0.16±0.02	0.05±0.01	0.23±0.06	0.003±0.008	1.24±0.65	0.33±0.11	173±44
	中剂量组	0.08±0.01	0.52±0.09	0.16±0.01	0.06±0.01	0.24±0.05	0.000±0.000	1.80±0.69	0.44±0.15	175±42
	高剂量组	0.09±0.01	0.54±0.09	0.15±0.01	0.06±0.01	0.21±0.03	0.000±0.000	1.42±0.69	0.50±0.23	170±26
给药后 210min (n=6)	空白对照组	0.08±0.02	0.51±0.09	0.14±0.02	0.06±0.01	0.21±0.02	0.002±0.004	1.21±0.43	0.45±0.17	182±24
	低剂量组	0.07±0.01	0.50±0.09	0.16±0.03	0.05±0.01	0.23±0.07	0.003±0.008	1.26±0.67	0.33±0.11	172±44
	中剂量组	0.08±0.01	0.52±0.08	0.16±0.01	0.06±0.01	0.24±0.05	0.002±0.004	1.83±0.77	0.43±0.13	175±48
	高剂量组	0.08±0.01	0.54±0.09	0.14±0.02	0.06±0.01	0.20±0.02	0.000±0.000	1.42±0.69	0.51±0.24	178±29
给药后 240min (n=6)	空白对照组	0.07±0.02	0.54±0.12	0.14±0.02	0.06±0.01	0.22±0.04	0.000±0.000	1.28±0.46	0.48±0.19	178±29*
	低剂量组	0.08±0.01	0.49±0.10	0.16±0.03	0.06±0.01	0.24±0.08	0.005±0.010	1.32±0.76	0.33±0.13	171±44
	中剂量组	0.08±0.01	0.51±0.09	0.16±0.02	0.06±0.02	0.24±0.06	0.003±0.008	1.86±0.81	0.42±0.13	173±54
	高剂量组	0.08±0.02	0.55±0.08	0.14±0.02	0.06±0.02	0.20±0.02	0.000±0.000	1.45±0.67	0.52±0.24	178±39

注：药前采集 1 次基础值，*/**与组内比较有显著性差异（p <0.05/0.01），△/△△与同期空白组比较有显著性差异（p <0.05/0.01）。

Notes:

Data were expressed as mean ± SD.

* $p < 0.05$ self-comparison with before administration.

△ $p < 0.05$ compared with the vehicle group.

Table S15 Effect of IAAS on blood pressure and respiratory parameters in anesthetized dogs

表 56　灯台叶碱提取物安全性药理血压呼吸检测数据统计结果（$\bar{x}$ ±SD）

测定时间	组别	收缩压 (mmHg)	舒张压 (mmHg)	平均压 (mmHg)	呼吸频率（次/ min）	呼吸深度（volt）
给药前 (n=6)	空白对照组	206±44	143±34	164±36	19±6.15	0.69±0.34
	低剂量组	200±49	138±46	158±48	14±5.11	0.81±0.56
	中剂量组	195±34	136±17	156±22	17±8.50	0.70±0.20
	高剂量组	208±36	141±33	163±34	12±4.68	0.70±0.17
给药后 30min (n=6)	空白对照组	198±45	136±33	155±36	18±5.99	0.67±0.34
	低剂量组	183±63	124±54	143±59	19±10.65	0.92±0.56
	中剂量组	198±31	138±20	160±22	18±8.34	0.70±0.18
	高剂量组	200±34	134±28	157±31	14±4.69	0.71±0.23
给药后 60min (n=6)	空白对照组	192±45	128±31	149±35	20±6	0.70±0.34
	低剂量组	178±62	123±56	141±58	20±10	0.91±0.60
	中剂量组	200±30	138±19	158±21	18±9	0.73±0.18
	高剂量组	180±45	120±34	140±37	13±3	0.72±0.19
给药后 90min (n=6)	空白对照组	187±51	124±33	144±37	20±8	0.66±0.37
	低剂量组	174±62	116±57	135±58	20±11	0.86±0.54
	中剂量组	202±33	137±19	157±22	19±9	0.78±0.15
	高剂量组	175±42	113±35	134±36	13±2	0.72±0.18
给药后 120min (n=6)	空白对照组	186±49	121±34	143±36	20±8	0.62±0.36
	低剂量组	167±64	108±59	128±61	19±12	0.85±0.55
	中剂量组	205±36	136±21	159±24	20±9	0.81±0.19
	高剂量组	172±47	111±33	132±37	12±2	0.69±0.17
给药后 150min (n=6)	空白对照组	190±49	125±33	146±36	18±8	0.59±0.38
	低剂量组	161±68	106±61	124±63	18±12	0.84±0.58
	中剂量组	204±33	135±20	157±22	20±10	0.74±0.16
	高剂量组	179±48	116±39	138±41	12±1	0.66±0.17
给药后 180min (n=6)	空白对照组	192±48	127±34	147±38	19±6	0.58±0.38
	低剂量组	160±68	104±61	124±64	19±10	0.82±0.57
	中剂量组	204±30	132±19	156±21	20±10	0.74±0.17
	高剂量组	181±45	118±36	140±39	13±2	0.64±0.18
给药后 210min (n=6)	空白对照组	189±44	124±28	145±33	20±6	0.59±0.38
	低剂量组	160±71	104±65	122±69	20±11	0.79±0.55
	中剂量组	203±29	133±14	158±19	20±10	0.74±0.19
	高剂量组	186±42	122±29	144±32	13±2	0.63±0.19
给药后 240min (n=6)	空白对照组	184±47	121±30	142±36	19±7	0.59±0.38
	低剂量组	159±73	104±66	123±69	18±9	0.78±0.55
	中剂量组	203±28	135±20	158±21	20±10	0.73±0.18
	高剂量组	189±43	124±30	145±33	13±3	0.64±0.20

Notes:

Blood pressure included systolic blood pressure, diastolic blood pressure and mean arterial pressure.

Respiratory parameters included respiration frequency and depth

Data were expressed as mean ± SD.

$p > 0.05$, there was no significant difference between the groups.

Reference

Adams, G.L., Carroll, P.J., Smith, A.B., 2012. Total Synthesis of (+)-Scholarisine A. J. Am. Chem. Soc. 134, 4037-4040.

Adams, G.L., Carroll, P.J., Smith, A.B., 2013. Access to the Akuammiline Family of Alkaloids: Total Synthesis of (+)-Scholarisine A. J. Am. Chem. Soc. 135, 519-528.

Cai, X.H., Bao, M.F., Zhang, Y., Zeng, C.X., Liu, Y.P., Luo, X.D., 2011. A New Type of Monoterpenoid Indole Alkaloid Precursor from *Alstonia rostrata*. Org. Lett. 13, 3568-3571.

Cai, X.H., Du, Z.Z., Luo, X.D., 2007. Unique Monoterpenoid Indole Alkaloids from *Alstonia scholaris*. Org. Lett. 9, 1817-1820.

Cai, X.H., Tan, Q.G., Liu, Y.P., Feng, T., Du, Z.Z., Li, W.Q., Luo, X.D., 2008. A cage-monoterpene indole alkaloid from *Alstonia scholaris*. Org. Lett. 10, 577-580.

Gerfaud, T., Xie, C.S., Neuville, L., Zhu, J.P., 2011. Protecting-Group-Free Total Synthesis of (E)- and (Z)-Alstoscholarine. Angew. Chem., Int. Ed. 50, 3954-3957.

Higuchi, K., Suzuki, S., Ueda, R., Oshima, N., Kobayashi, E., Tayu, M., Kawasaki, T., 2015. Asymmetric Total Synthesis of (−)-Leuconoxine via Chiral Phosphoric Acid Catalyzed Desymmetrization of a Prochiral Diester. Org. Lett. 17, 154-157.

Liang, X., Jiang, S.Z., Wei, K., Yang, Y.R., 2016. Enantioselective Total Synthesis of (-)-Alstoscholarisine A. J. Am. Chem. Soc. 138, 2560-2562.

Mason, J.D., Weinreb, S.M., 2018. Synthesis of Alstoscholarisines A-E, Monoterpene Indole Alkaloids with Modulating Effects on Neural Stem Cells. The Journal of Organic Chemistry 83, 5877-5896.

Moreno, J., Picazo, E., Morrill, L.A., Smith, J.M., Garg, N.K., 2016. Enantioselective Total Syntheses of Akuammiline Alkaloids (+)-Strictamine, (-)-2(S)-Cathafoline, and (-)-Aspidophylline A. J. Am. Chem. Soc. 138, 1162-1165.

Pan, Z.Q., Qin, X.J., Liu, Y.P., Wu, T., Luo, X.D., Xia, C.F., 2016. Alstoscholarisines H-J, indole alkaloids from *Alstonia scholaris*: structural evaluation and bioinspired synthesis of alstoscholarisine H. Org. Lett. 18, 654-657.

Ren, W.W., Wang, Q., Zhu, J.P., 2016. Total Synthesis of (±)-Strictamine. Angew. Chem., Int. Ed. 55, 3500-3503.

Smith, J.M., Moreno, J., Boal, B.W., Garg, N.K., 2014. Total Synthesis of the Akuammiline Alkaloid Picrinine. J. Am. Chem. Soc. 136, 4504-4507.

Smith, J.M., Moreno, J., Boal, B.W., Garg, N.K., 2015. Fischer Indolizations as a Strategic Platform for the Total Synthesis of Picrinine. J. Org. Chem. 80, 8954-8967.

Smith, M.W., Snyder, S.A., 2013. A Concise Total Synthesis of (+)-Scholarisine A Empowered by a Unique C-H Arylation. J. Am. Chem. Soc. 135, 12964-12967.

Umehara, A., Ueda, H., Tokuyama, H., 2014. Total syntheses of leuconoxine, leuconodine B, and melodinine E by oxidative cyclic aminal formation and diastereoselective ring-closing metathesis. Org. Lett. 16, 2526-2529.

Wang, D., Hou, M., Ji, Y., Gao, S.H., 2017. Total Synthesis of Scholarisine K and Alstolactine A. Org. Lett. 19, 1922-1925.

Watanabe, T., Kato, N., Umezawa, N., Higuchi, T., 2013. Synthesis of the Carbon Framework of Scholarisine A by Intramolecular Oxidative Coupling. Chem. Eur. J. 19, 4255-4261.

Xu, Z.R., Wang, Q., Zhu, J.P., 2015. Total Syntheses of (-)-Mersicarpine, (-)-Scholarisine G, (+)-Melodinine E, (-)-Leuconoxine, (-)-Leuconolam, (-)-Leuconodine A, (+)-Leuconodine F, and (-)-Leuconodine C: Self-Induced Diastereomeric Anisochronism (SIDA) Phenomenon for Scholarisine G and Leuconodines A and C. J. Am. Chem. Soc. 137, 6712-6724.

Yang, X.W., Qin, X.J., Zhao, Y.L., Lunga, P.K., Li, X.N., Jiang, S.Z., Cheng, G.G., Liu, Y.P., Luo, X.D., 2014a. Alstolactines A-C, novel monoterpenoid indole alkaloids from *Alstonia scholaris*. Tetrahedron Lett. 55, 4593-4596.

Yang, X.W., Song, C.W., Zhang, Y., Khan, A., Jiang, L.P., Chen, Y.B., Liu, Y.P., Luo, X.D., 2015. Alstoscholarisines F and G, two unusual monoterpenoid indole alkaloids from the leaves of *Alstonia scholaris*. Tetrahedron Lett. 56, 6715-6718.

Yang, X.W., Yang, C.P., Jiang, L.P., Qin, X.J., Liu, Y.P., Shen, Q.S., Chen, Y.B., Luo, X.D., 2014b. Indole Alkaloids with

New Skeleton Activating Neural Stem Cells. Org. Lett. 16, 5808-5811.
Yang, Y., Bai, Y., Sun, S., Dai, M., 2014c. Biosynthetically inspired divergent approach to monoterpene indole alkaloids: total synthesis of mersicarpine, leuconodines B and D, leuconoxine, melodinine E, leuconolam, and rhazinilam. Org. Lett. 16, 6216-6219.
